# *CCR5*-Δ32 biology, gene editing, and warnings for the future of CRISPR-Cas9 as a human and humane gene editing tool

**DOI:** 10.1186/s13578-020-00410-6

**Published:** 2020-03-30

**Authors:** MengMeng Xu

**Affiliations:** Department of Pediatrics, Morgan Stanley Children’s Hospital, Columbia University, 3959 Broadway, New York, NY 10032 USA

**Keywords:** *CCR5-Δ32*, Human genome editing, HIV infection, CRISPR-Cas

## Abstract

**Background:**

Biomedical technologies have not just improved human health but also assisted in the creation of human life. Since the first birth of a healthy baby by in vitro fertilization (IVF) 40 years ago, IVF has been the mainstay treatment for couples struggling with infertility. This technology, in addition to increasingly accessible genetic testing, has made it possible for countless couples to have children. Since CRISPR-Cas9 gene editing was described in 2015, its potential for targeting genetic diseases has been much anticipated. However, the potential of using CRISPR-Cas9 for human germline modification has led to many fears of “designer babies” and widespread concerns for the impact of this technology on human evolution and its implications in Social Darwinism. In addition to these ethical/moral concerns, there remain many unknowns about CRISPR-Cas9 technology and endless unanticipated consequence to gene editing.

**Methods:**

In this paper, we analyze the current progresses of CRISPR-Cas9 technology and discuss the theoretical advantages of certain allelic variances in the C-C chemokine receptor 5 gene (CCR5) in the setting of recent ethical/moral concerns regarding gene editing using the CRISPR-Cas9 system.

**Results:**

These uncertainties have been highlighted recently by the birth of Chinese twins whose C-C chemokine receptor 5 (*CCR5*) gene had been inactivated via CRISPR-Cas9 to be theoretically protective against HIV infection. CCR5 signaling is critical for the successful infection of human immunodeficiency virus (HIV) and people with homozygous inactivating *CCR5-Δ32* mutations have been shown to be protected against HIV infection. Those with the *CCR5*-*Δ32/Δ32* mutation also have greater neuroplasticity, allowing for improved recovery from neurological trauma, and decreased Chagas cardiomyopathy. However, the *CCR5*-*Δ32/Δ32* mutation has also been associated with earlier clinical manifestations for West Nile infection, ambiguous effects on osteoclast function, and a four-fold increased mortality from influenza infection. These detrimental health impacts, in addition to the confounding factor that these CRISPR babies do not carry this exact *CCR5-Δ32/Δ32* mutation, lead to many questions regarding the children’s future health and the moral conundrum of their birth. The creation and birth of these babies was not completed with any scientific, ethical, or governmental oversight, which has spurned the acceleration of talks regarding global regulations for human genetic editing.

**Conclusions:**

Although we can try to regulate for ethical, health-related only use of this technology, moral and governmental oversights need to be supplemented by technical regulations. For instance, whole genome sequencing needs to be used to eliminate off-target mutations that could affect the health and safety of infants born to this process. Like Pandora’s Box, we cannot pretend to forget CRISPR-Cas9 technology, all we can do is ensure a safe, moral, and equitable used of this technology.

As the most efficient and precise genome editing tool available, CRISPR-Cas9 technology presents a powerful and lost-cost method of genetic editing that has never been available before. The availability of this technique has radically changed the biomedical field and has the potential to radically alter human healthcare [1–3]. It has made in vitro modeling of human mutations possible, increased the speed of genetically engineered animal models, and made the treatment of genetic diseases more than a pipedream. In fact, a pilot clinical trial in sickle cell anemia just reported promising preliminary results in the first patient ever treated with CRISPR-Cas9 gene-therapy [4] and there are multiple other ongoing trials assessing gene therapy in hematologic disease [5]. The power of CRISPR-Cas9 technology is not limited to the correction of disease-causing genetic mutations, but also being considered as a method for taking advantage of genetic traits inherent in some populations. For instance, the C-C chemokine receptor 5 (*CCR5*) Δ32 mutation found in ~ 11% of northern Europeans is known to protect against HIV infection. Last year, twin Chinese girls were engineered by CRISPR-Cas9 to carry a *CCR5* gene with similar properties to *CCR5*-Δ32, specifically to be resistant to HIV. The announcement of these unexpected births has highlighted the fear of a new era of eugenics brought on by CRISPR-Cas9. Here we discuss the protective and detrimental effects of this mutation and contribute to the ongoing moral, philosophical, and regulational conversation with considerations regarding the technical safety of CRISPR-Cas9 technique in humans.

The *CCR5* gene was first identified in 1977 [6] but did not become a subject of great public interest until 2009, when an HIV positive individual transplanted with bone marrow from a donor with a homozygous *CCR5*-Δ32 mutation, became HIV negative despite stopping anti-retroviral (ARV) therapy [7]. This seminal clinical case study was founded on decades of work showing CCR5’s role as a co-stimulator in T-cell function, activation, and the production of antigen specific T-cells [8]. These studies showed the *CCR5*-Δ32 mutation to cause deletion of 32-base pairs in *CCR5*, leading to non-functional expression of this gene that does not localize to the cell surface. These mechanistic findings along with the discovery of CCR5 as a necessary co-receptor for entry of macrophage tropic HIV strains [9, 10] led to increased interest in this gene as a target for HIV treatment and other immunological processes.

*CCR5* deletions have also been shown to provide protection against other pathogens, including smallpox and flaviviruses such as dengue, Zika, and West Nile virus [11]. In fact, smallpox endemics in Europe are believed to be the selective pressure that led to an increased presence of the allele in European populations [11]. *CCR5* deletion was also found to be protective against non-viral infections. Early reports have found the *CCR5-Δ32* deletion to be protective against inflammatory cardiomyopathy in patients with chronic Chagas’ disease [12]. This result was recently disputed in a polymorphism analysis between wild-type, heterozygous, and homozygous Chaga’s disease patients [13]. However, a Brazilian genetic polymorphism study of *CCR1*, *CCR5*, and their ligands *CCL2* and *CCL5*, respectively, found CCL5-CCR1 to be the target for immune-stimulation from *Trypanosoma cruzi* infection. Certain variants of CCL5-CCR1 were subsequently found to be significantly protective against Chagas’s disease [14]. Outside of the infectious disease realm, *CCR5* has also been found to be involved in neuronal recovery from stroke and traumatic brain injury (TBI) through upregulation of CREB (cAMP response element-binding protein) and DLK (Delta-like protein 1) signaling [15]. Joy et al. first identified the expression of CCR5 in cortical neurons after stroke and later discovered neuronal knockdown of *CCR5* to result in enhanced cortical projections during regeneration and preservation of dendritic spines [15]. These in vitro findings were subsequently confirmed as clinically significant in an analysis of 1,563 stroke patients (300 CCR5-Δ32 carriers vs 1265 non-carriers) in the Tel Aviv Brain Acute Stroke Cohort (TABASCO). Patients with Δ32/Δ32 loss-of-function mutation *CCR5* recovered significantly faster from stroke with improved measures of memory, verbal function, and attention- indicating improved neuronal plasticity [15]. While *CCR5* is clinically relevant in this wide variety of diseases, its importance in HIV infection has been the most studied in the clinical setting.

As a cell membrane integrated protein with seven transmembrane segments and an eighth α-helix parallel to the plasma membrane, CCR5 presents on the cell surface and functions in tandem with CD4-recptors as the initial co-docking site for the HIV PG120-PG41 surface protein. This initial association between the HIV PG120-PG41, CCR5, and CD4-receptors allows for the initial viral invasion and subsequent infection and replication (Fig. 1a). The essential binding site on CCR5 for HIV PG120-PG41 is known as 2D7. It is located on the third extracellular element (second loop) of the membrane integrated CCR5 and works in tandem with the PA12 binding site and the G protein linkage domains found on the first extra-cellular element of CCR5. The CCR5-Δ32 mutation, describes a 32 base pair deletion just before the 2D7 structural loop. This results in the creation of a premature stop codon, and thus, the absence of the 2D7 loop necessary for HIV viral binding, but preserves the PA12 binding site (Fig. 2). This mutation hampers HIV binding two-fold: by removing the necessary 2D7 binding domain and by rendering the protein cytosolic. Around 10% of the European population have paired missense mutations C20S and C178R or C101X and FS299, collectively known as *CCR5*-Δ32, which protects against HIV infection by inhibiting the initial viral docking process (Fig. 1b) [16, 17].

**Fig. 1:**
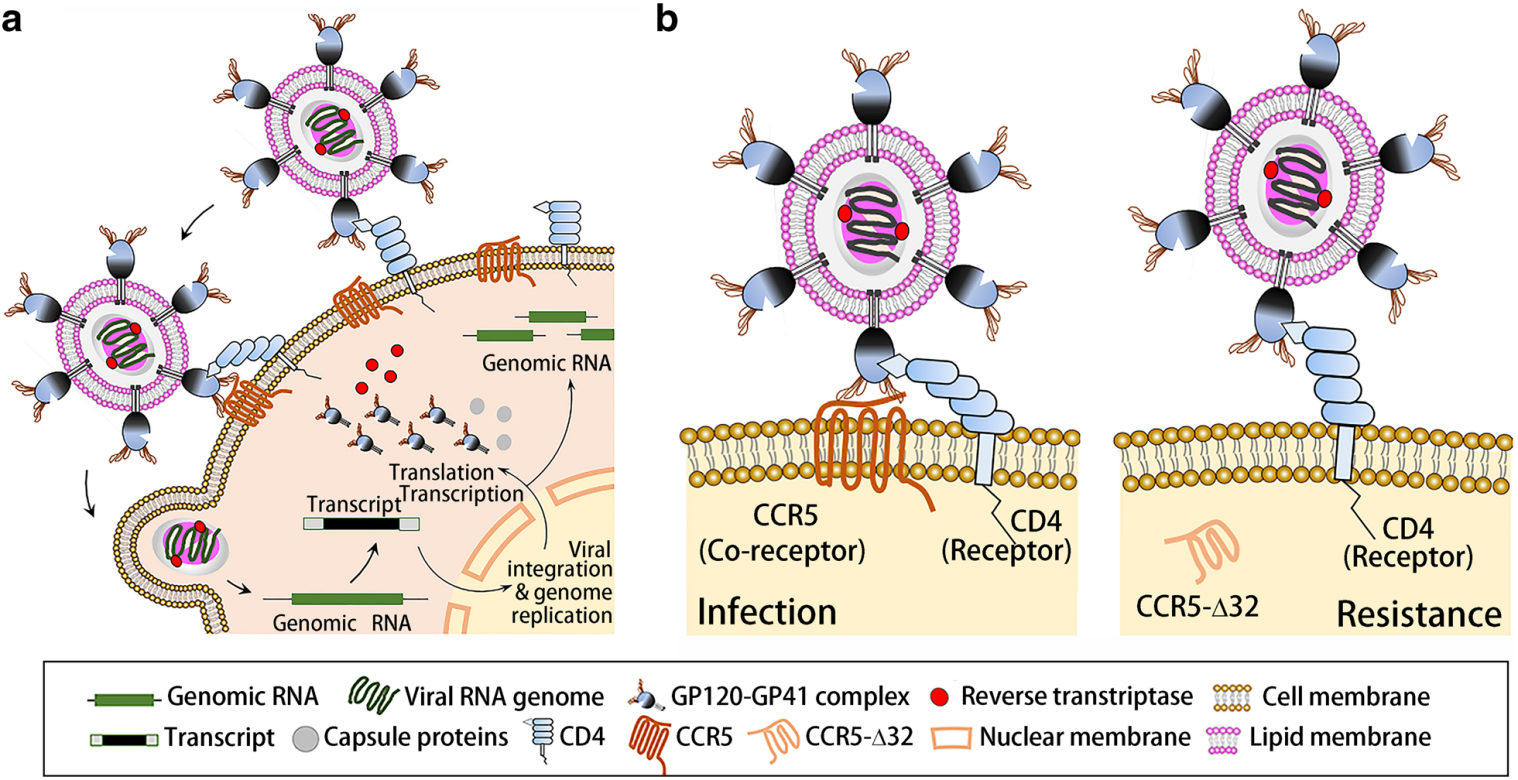
The HIV infection process (**a**): The HIV GP-120 first associates with both the CD4 and CCR5 on the surface of a cell, which is the first step in viral invasion and further viral replication. Molecular mechanism of CCR5 in HIV infection and the protective effect of cytoplasmic CCR5-Δ32 against HIV-1 infection (**b**)

**Fig. 2:**
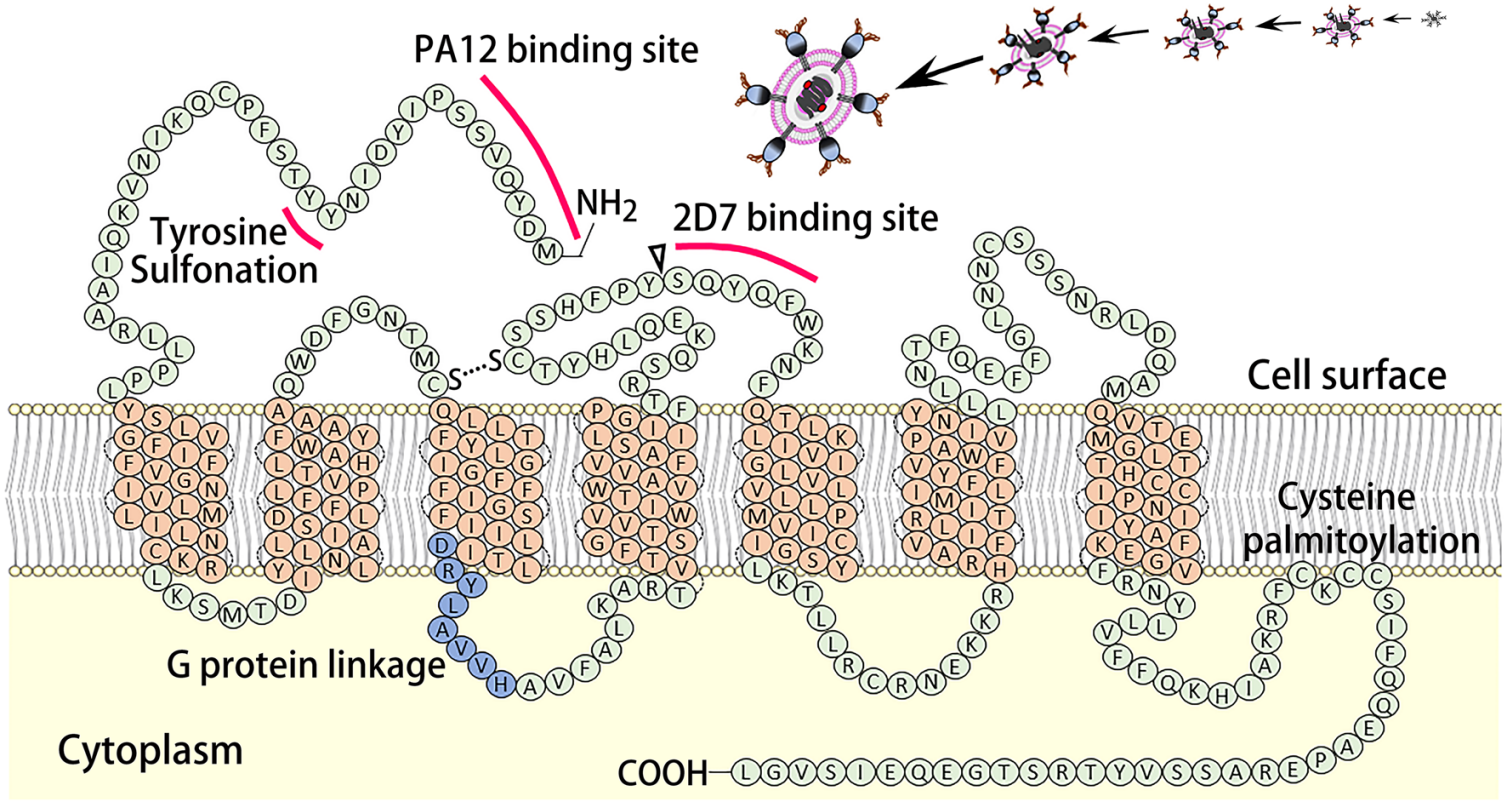
The structure of membrane integrated CCR5. The elements important in HIV binding and structure (PA12 binding site and 2D7 binding site, and sites of tyrosine sulfonation and G-protein linkage) are highlighted. The CCR5-Δ32 deletion site is denoted with a triangle and found just before the 2D7 binding site. Mutation at this site results in a premature stop codon, and thus the deletion of all protein structures after this location, resulting in the loss of the 2D7 binding site and a cytosolic CCR5

Ever since the theoretical protection of *CCR5*-Δ32/Δ32 against HIV was clinically supported by the cure of a HIV-positive patient transplanted with bone marrow from a homozygous *CCR5*-Δ32 donor [7], the potential for *CCR5*-Δ32 as a curative therapy for HIV has been greatly debated and anticipated [8, 17, 18]. However, most controlled and regulated studies are still in the pre-clinical phase using human stem cells or mouse models. The Deng group established a CRISPR/Cas9 gene editing system in human CD34^+^ hematopoietic stem cells (HSPCs) which allowed for long-term *CCR5* ablation. Mice transplanted with these CCR5-deleted HSPCs exhibited lasting HIV-1 resistance in vivo [19]. Another study found editing of co-receptors CCR5 and CXCR4 by CRISPR-Cas9 to protect CD4+ T cells from HIV-1 infection in vitro [20]. Although another group was able to successfully transplant and achieve long-term engraftment of CRISPR-edited HSPCs into a patient, they were only able to disrupt 5% of CCR5 function. This unexpected result hinted at unanticipated factors in in vivo editing, thus halting the study for fear of harm to patient health [21, 22]. Despite the lack of complete understanding of the *CCR5* gene and incomplete pre-clinical testing proving *CCR5* gene manipulation to be benign, some have already jumped ahead to human genome manipulation. Last year, Jiankui He, a researcher at the Southern University of Science and Technology in Guandong, China announced the birth of twins whose genomes he had manipulated by CRISPR-Cas9 to have non-functional *CCR5.* This editing was made in an effort to protect the infants against HIV infection. This unregulated experiment immediately generated massive concern over the moral impact of this human experiment and earned universal condemnation for advancing to human experimentation without adequate safety precautions and assessments.

While the use of CRISPR-Cas9 technology as a eugenics tool is morally confounding and difficult to justify given the human health, evolution, and social equality implications; it is naïve to say that CRISPR-Cas9 will not be used by futures parents and scientist to give an advantageous foundation to their children. Thus, the best course of action that global summits on genome editing can produce are exact allowances and restrictions for genome editing and specific punishments for both the researcher and the local/federal governments responsible for enforcing regulations. Inherited disease caused by specific point mutations may be the most realistic targets for germline alternation. For instance, correcting the point mutation causing the glutamine to valine mutation in sickle cell disease could free future generation from the constant threat of pain crises and eliminate the risk of acute chest and stroke that often claim these patients’ lives. However, even in these clear-cut cases we still need further data on the exact time period during which germline alteration is safe for the embryo. However, to ensure at least the methodological safety of using CRISPR-Cas9 in humans, two technical aspects must be met: total understanding of the gene being altered and complete control over off target effects of CRISPR-Cas9 editing. Editing of *CCR5* does not fit the first requirement as those homozygous for the *CCR5*-Δ32 mutation have unexpected negative effects such as earlier clinical manifestations for West Nile infection [23], four-fold likelihood of mortality from influenza infections [24], and disadvantageous osteoclast function [25]. In addition, multiple publications have reported unexpected off-target mutations generated by CRISPR-Cas9. Although one retracted publication demonstrated few unexpected mutational events [26], one study found rare but notable mutations [27], several others found large deletions [28, 29], while another found unexplainable complex deletions and insertions in mice generated by CRISPR-Cas9 [30]. As such, the CCR5 twins need to be monitored both for possible known effects, such as an increased susceptibility to influenza infection, abnormal bone growth and other immunological conditions, and also require close monitoring of their general growth and development for unanticipated effects.

Even should these unknowns be overcome, there may still be small deletions or insertions that cause deleterious frame-shift mutations, or rarer effects we have yet to identify. As such, the only way to ensure the coding fidelity of edited cells is by sequencing the full genome of each edited cell in comparison to parents’ genomes. This safety check itself will require further technological development allowing for rapid, inexpensive whole-genome sequencing and analysis while in the narrow window of implantable embryos. Even these precautions would not account for the epigenetic factors that may impact growth and development. Should complications from these identified elements be resolved, there are still a myriad of unknown factors in CRISPR-Cas9 technology that should present an independent technological precaution against human genetic editing regardless of the moral/ethical conundrum (Fig. 3). We suggest that there needs to be a more vigorous and annual global debate to established the specific mutations on which human gene-editing research should be allowed, and that these genes be limited to those what would solve clear clinical problems (i.e*.* sickle cell disease, other diseases with known mutational causes). Ideally, such a body of experts would also be able to advise a multinational consortium such as the United Nations on the appropriate punitive and incentive actions necessary to dissuade individuals and institutions from supporting unsanctioned human genome editing.

**Fig. 3:**
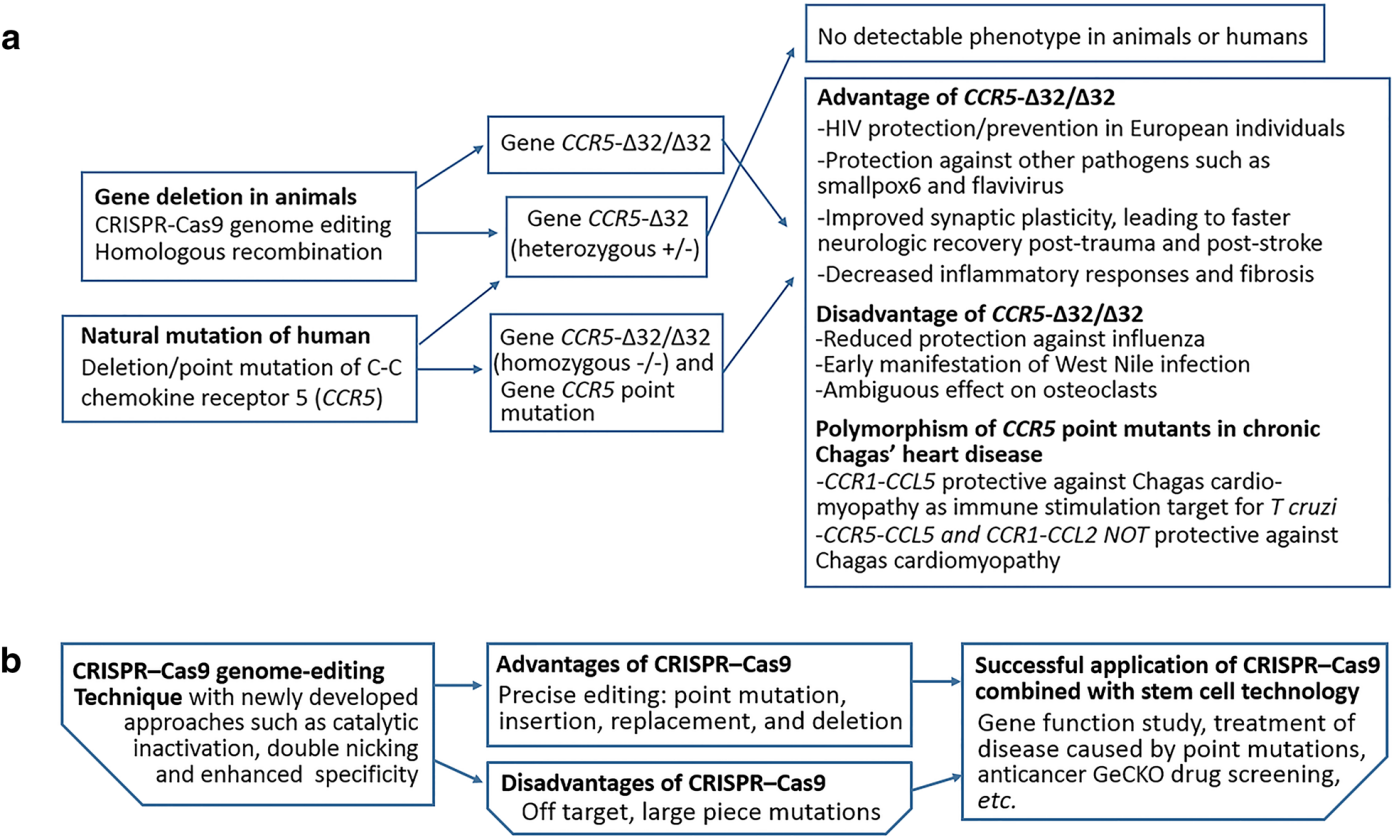
Advantages and disadvantages of *CCR5*-Δ32/Δ32 (**a**) and CRISPR-Cas9 (**b**) genome editing

## Data Availability

Not applicable.

## Abbreviations

ARV: Anti-retroviral
CCR5: C-C chemokine receptor 5
CCL2: C-C motif chemokine ligand 2
CREB: cAMP response element-binding protein
CRISPR: Clustered regularly interspaced short palindromic repeats
Cas9: *CRISPR*-associated protein-9 nuclease
CXCR4: C-X-C chemokine receptor type 4
DLK1: Delta-like protein 1
GP120: Glycoprotein 120, viral envelope glycoprotein 120
GP41: Glycoprotein 41, viral envelope glycoprotein 41
HSPC: Hematopoietic stem cell
HIV: Human immunodeficiency virus
IVF: In vitro fertilization
TABASCO: Tel Aviv Brain Acute Stroke Cohort
TBI: Traumatic brain injury
